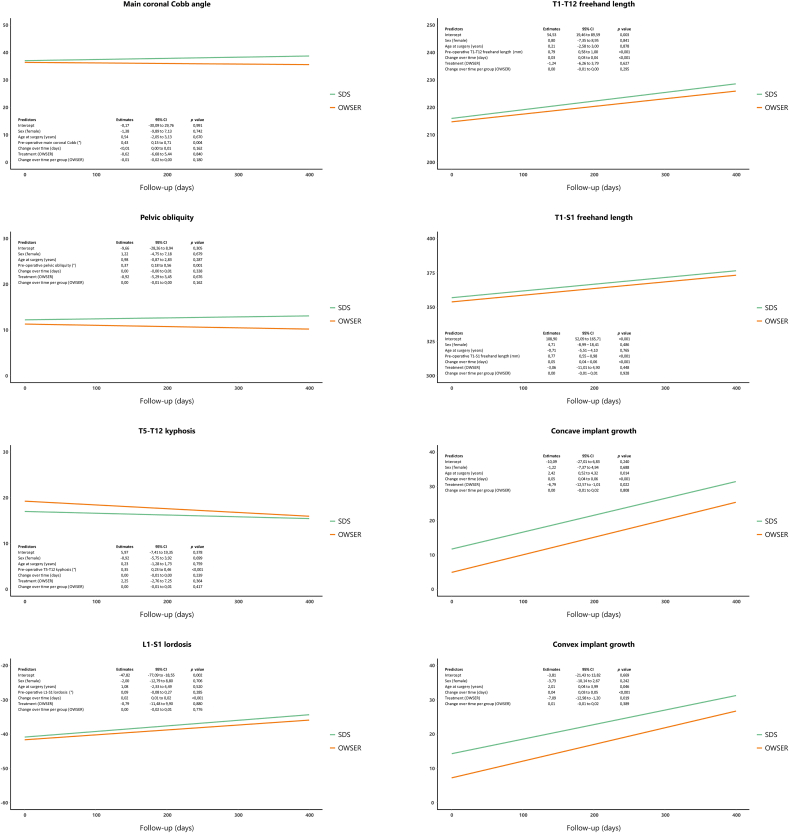# One Year Results of the Randomized BiPOWR Trial Comparing the Spring Distraction System (SDS) and the One Way Self-Expanding Rod (OWSER) for the Correction of Neuromuscular and Syndromic Early Onset Scoliosis

**DOI:** 10.1016/j.jposna.2025.100180

**Published:** 2025-03-27

**Authors:** Justin V.C. Lemans, Casper S. Tabeling, Agnita Stadhouder, Jeroen J.M. Renkens, E. Pauline Scholten, Hilde W. Stempels, Lotfi Miladi, René M. Castelein, Moyo C. Kruyt

**Affiliations:** 1 UMC Utrecht, Utrecht, the Netherlands; 2 Amsterdam UMC, Amsterdam, the Netherlands; 3 Erasmus MC, Rotterdam, the Netherlands; 4 Hôpital Necker-Enfants Malades, Paris, France

**Keywords:** BiPOWR, Early onset scoliosis, Neuromuscular, Spring distraction system, One way self-expanding rod

## Abstract

**Background:**

Current “growth-friendly” implants for treatment of Early Onset Scoliosis (EOS) have limitations that reduce their efficacy and cost-effectiveness. Recently, two systems have been developed that mitigate many of these limitations, the Spring Distraction System (SDS) and the One Way Self-Expanding Rod (OWSER). The purpose of the multicenter BiPOWR trial was to compare 1-year efficacy and -safety of both strategies in the treatment of neuromuscular or syndromic EOS.

**Methods:**

Non-ambulant, neuromuscular/syndromic EOS patients were included in three academic hospitals. They were randomized to treatment with SDS or OWSER and were blinded until after surgery. Outcomes were coronal curve, spinal growth and the occurrence of (serious) adverse events ((S)AEs). In addition, spinal growth and implant lengthening were calculated. Data were collected pre-operatively, immediately post-operatively, and at 1-, 3-, 6-, and 12-month follow-up.

**Results:**

Thirty patients were included. Two patients passed away during follow-up, and these patients were replaced. All collected data were used for analysis. Mean age at surgery was 9.0 years, and 20/30 patients were male. Mean coronal curve decreased from 74.9° pre-operatively, to 37.6° post-operatively, remaining stable at 37.7° at the 1-year follow-up, with no group differences. T1-T12 length increased by 18 mm/year for SDS and 9 mm/year for OWSER. For T1-S1 length, this was 26 mm/year (SDS) and 18 mm/year (OWSER). Five (S)AEs occurred in the SDS group and 11 (S)AEs in the OWSER group. Two SDS patients passed away, unrelated to the surgery or implant. One (S)AE in the SDS group and 6 (S)AEs in the OWSER group were implant-related.

**Conclusions:**

The SDS and the OWSER achieved coronal curve correction of 50%, which was maintained at 1-year follow-up. Spinal length increase was excellent for both systems. The (S)AE rate was 30%/patient/year for SDS and 78%/patient/year for OWSER.

**Key Concepts:**

(1) The current study is the first RCT that compares two “growth-friendly” implants in a neuromuscular early onset scoliosis (EOS) population.
(2) The Spring Distraction System (SDS) and the One Way Self-Expanding Rod (OWSER) both achieve around 50% curve correction which is maintained at 1 year follow-up.
(3) Both systems achieve excellent T1-T12- and T1-S1 height increase, without the need for repetitive lengthenings.
(4) The (S)AE rate of SDS was 30%/patient/year. For OWSER, the (S)AE rate was 78%/patient/year.

**Study design:**

RCT

**Level of Evidence:**

Level 1

## Introduction

Early onset scoliosis (EOS) represents a complex deformity of the spine and trunk. Deformities can cause significant health problems, particularly pulmonary compromise, which emphasizes importance on early intervention [[1], [2], [3]]. Successful management relies on preventing the progression of the spinal deformity, allowing growth of the spine and trunk and lung development to maximize pulmonary function with a minimal amount of complications [3]. Conservative treatment such as casting or bracing are commonly used for early intervention [4]. However, especially in neuromuscular or syndromic EOS, these techniques are at best able to delay surgery and have many drawbacks [5,6]. Surgical treatment of the growing spine is complex. In the past, the standard approach was a long-segment spinal fusion, often to the pelvis, resulting in growth arrest with the risk of underdevelopment of the lungs [7]. In addition, posterior fusion alone may lead to progression of the spinal deformity secondary to the remaining anterior growth of the spine, known as the “crankshaft” phenomenon [8]. To address these issues, growth-friendly procedures have been developed such as the traditional growing rod (TGR) and magnetically controlled growing rod (MCGR) [9,10]. While both are able to control the spinal deformity and allow spinal growth by distraction, the complication rate is high and results can still be improved [11,12]. For the TGR, the most obvious limitation is the need for repetitive surgical lengthenings (generally every 6 months), increasing both the risk of complications and the anesthetic burden on the child [13,14]. The MCGR allows for more frequent lengthening without surgery. However, more frequent returns to the outpatient clinic imposes a psychological burden on the patients and their parents [15]. Besides these inherent limitations, both systems have high mechanical (i.e. implant-related) complication rates which lead to many unplanned reoperations and diminished length gain [13,[16], [17], [18], [19], [20]]. For MCGR, rod metallosis due to high frictional forces was a reason to temporarily withdraw it from the market and currently, the FDA has approved its use for no longer than two years of implantation, although many devices are implanted for much longer [[21], [22], [23], [24]]. Another important disadvantage of the ‘traditional’ systems is that lengthening is intermittent, rigid and abrupt [19,25,26]. An ideal system provides continuous, dynamic lengthening of the spine without further interventions. To combat the limitations of TGR and MCGR while utilizing their advantages, two new “growth-friendly” systems were developed: the Spring Distraction System (SDS) and the One Way Self-Expanding Rod (OWSER). The SDS (not yet FDA approved) uses a compressed spring around a conventional rod to generate distraction forces. The OWSER (FDA approved and CE-marked as Nemost®; Euros, SAS, La Ciotat, France) uses a notched rod that can lengthen one-way with a split-ring retaining system. The most important advantages of the SDS and OWSER are that they can expand continuously, while maintaining deformity correction, without the necessity for surgical or outpatient clinic interventions. This, in combination with the less invasive method of implantation is especially useful for EOS patients with neuromuscular or syndromic etiologies, as these patients have increased risk of suffering from wound- and pulmonary complications following surgery [27,28]. The feasibility of both systems has been shown in previous studies, however, the patient populations and surgical strategies were quite heterogeneous [[29], [30], [31], [32]]. Moreover, the case series design in these studies may have led to selection bias. We therefore performed a randomized trial (BiPOWR) to compare 1-year efficacy and safety of both the SDS and OWSER in a similar group of neuromuscular or syndromic EOS patients.

## Methods

### Trial protocol, ethical review, and informed consent

The current study was approved by the Institutional Review Board of all three participating centers and was prospectively registered (Clinicaltrials.gov: NCT04021784). In addition, an elaborate study protocol was published previously. [33]. This study conformed to the CONSORT statement, the CONSORT checklist can be found in Supplement 1 [34]. All patients and parents/caregivers provided written informed consent before they were included into the trial.

### Study design

The current study is a prospective, multicenter, randomized limited-efficacy trial comparing the SDS and the OWSER in 3 academic hospitals in The Netherlands. Non-ambulatory neuromuscular or syndromic EOS patients were eligible for inclusion. Additional eligibility criteria are reported in Table 1. After inclusion, patients were randomized into either the SDS or OWSER group in a 1:1 ratio and their outcomes with respect to efficacy and safety were evaluated and compared during the first year of follow-up. A previous power calculation showed that fourteen patients in each arm were necessary to identify a 5° difference in coronal Cobb angle after 1 year with a power of 80% and an α of 0.05 [33]. Patients in each group were evaluated pre-operatively, immediately post-operatively, and at 1, 3, 6 and 12 months post-operatively.

**Table 1.** Eligibility criteria.

**Inclusion criteria**
Neuromuscular or syndromic early onset scoliosis (diagnosis before age 10)
Progressive early onset scoliosis with an indication for bipolar fixation extending to the pelvis
Non-ambulant patients
Age <12 years.
**Exclusion criteria**
Closed triradiate cartilage
Main curve proximal end vertebra at or above T3
Presence of skeletal dysplasia affecting growth (such as achondroplasia or spondyloepiphyseal dysplasia congenita)
Presence of disease that severely influences bone quality (such as osteogenesis imperfecta) or is associated with soft tissue weakness (such as Marfan syndrome, neurofibromatosis or Ehlers-Danlos syndrome)
Presence of active systemic disease
Congenital spinal anomaly of >5 vertebrae
Previous instrumented spinal surgery
Patients who cannot be followed for 1 year post-operatively

### Randomization and blinding

Before the trial commenced, a pseudorandom sequence of 28 numbers was created using a computer-generated permuted block design with random block sizes. This sequence was converted into allocation notes kept in double-sealed, opaque envelopes, which were sequentially opened after the surgery date was planned. The patient and his/her caregivers remained blinded until after surgery. This prevented the potential scenario in which patients/caregivers could withdraw from the study before surgery, if they were disappointed by the randomization result. Unblinding after surgery prevents this type of (selection) bias.

### Surgical procedure and treatment arms

All surgical procedures were performed using a less-invasive bipolar posterior approach and instrumentation with standard neuromonitoring [35]. To minimize surgical heterogeneity between centers, the senior author was present during all surgeries in all participating centers. Before the start of the study, the senior author had multiple years of experience using both the SDS implant as well as the OWSER implant. The distal anchor was created using iliosacral screws (Tanit®; Euros, SAS, La Ciotat, France) [36]. The proximal anchor for SDS consists of bilateral pedicle screws at 3 consecutive levels, typically T2-T4. For the OWSER, a series of proximal laminar- and pedicle hooks spanning 5 vertebrae was created (T2-T6).

**Figure 1:**
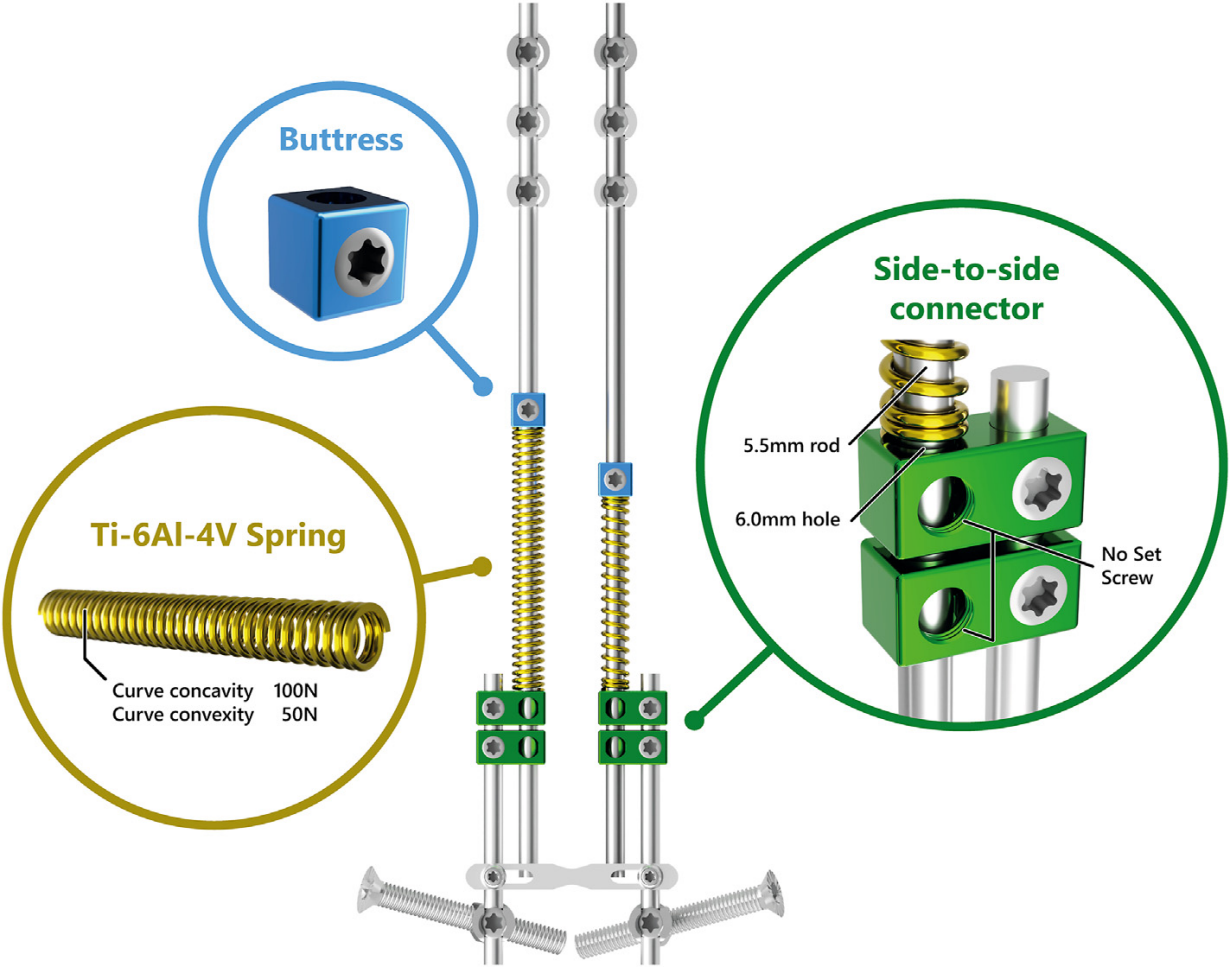
**Spring Distraction System**. The SDS consists of three components that are added to standard growing rods. It provides a continuous distraction force during follow-up, without the need for repeated lengthenings. In the BiPOWR trial, the distal anchors are iliosacral screws. **Green.** A side-to-side connector with one oversized hole through which a CoCr rod can slide freely. **Gold.** Ti6Al4V springs which can be compressed over the rod. **Blue.** The buttress compresses the spring against the side-to-side connector.

The SDS (Fig. 1) is an adjunct to 5.5 mm cobalt-chromium (CoCr) rods. It consists of three components: (1) a titanium (Ti6Al4V) spring placed over the rod, (2) two stacked oversized parallel connectors and (3) a buttress used to tension the springs. In neuromuscular EOS, the SDS is placed bilaterally. Around the concave rod, a 100 N spring (spring constant (*k*) = 1.33 N/mm, growing length 83 mm) was inserted, the convex rod received a weaker 50 N spring (*k* = 0.67 N/mm, growing length 83 mm). Both springs were maximally compressed. The rods that house the spring are left with 6–7 cm of residual rod length, which is the growth potential during follow-up. The parallel connectors were then secured to the distal anchor rods and the rod containing the spring was allowed to slide freely in the connectors.

**Figure 2:**
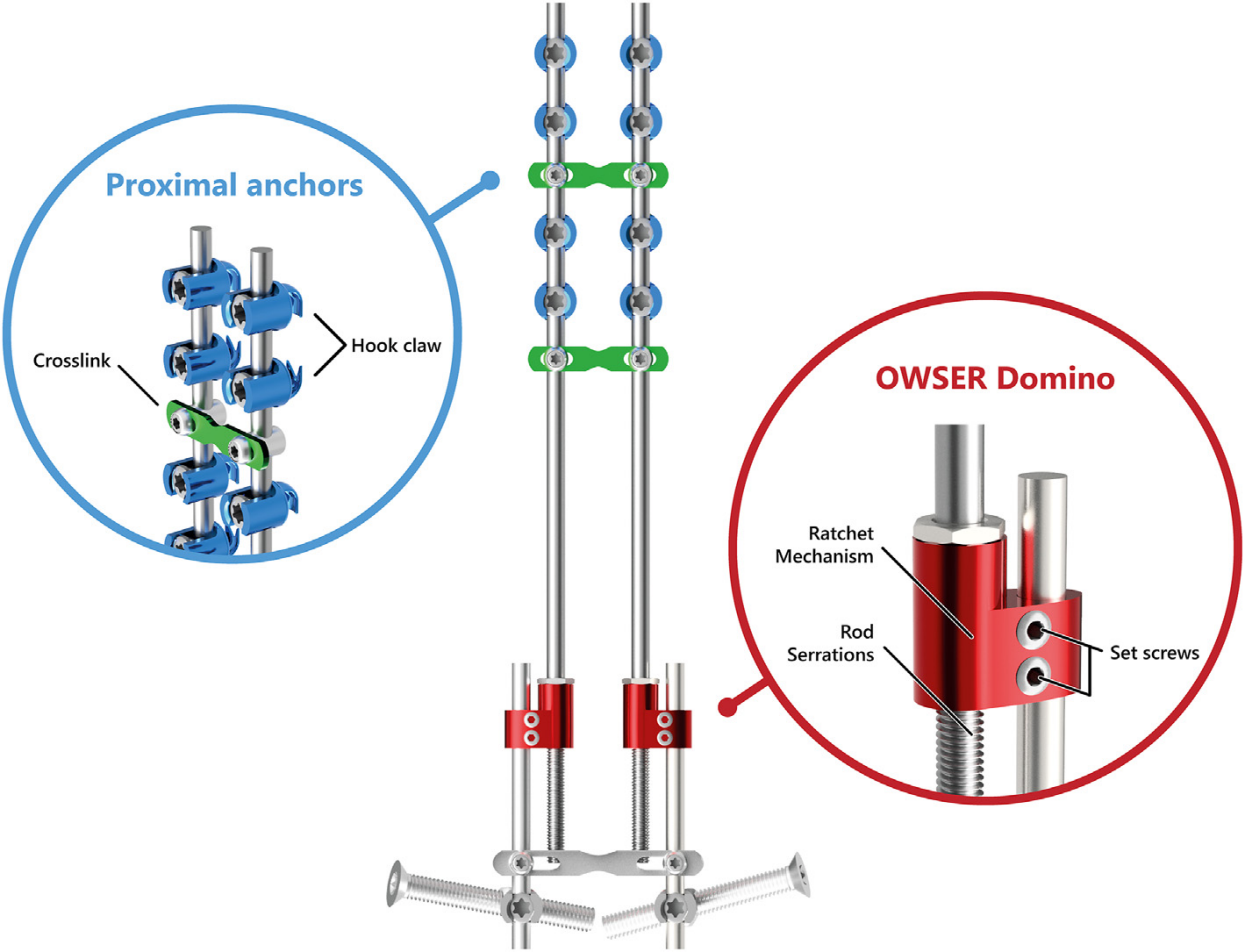
**One Way Self-expanding Rod.** The OWSER is a growing rod that passively lengthens one way as the spine grows. In the BiPOWR trial, the distal anchors are iliosacral screws instead of pedicle screws. **Blue.** the proximal fixation consists of hooks positioned in a claw configuration. Two crosslinks are added for torsional stability (green). **Red.** The growing domino, combined with a rod that is serrated across its distal length, allows for lengthening. The reserve length can be 50 mm or 80 mm long. Movement in the other direction is prevented by a split retaining ring system inside the domino.

The OWSER (Fig. 2) consists of two titanium components: (1) a 5.5 mm titanium long rod with a notched end, and (2) a sliding domino that passively migrates only one way across the notched segment. The sliding domino is fixated with a short rod to the distal anchor. Implant growth potential (i.e. the notched segment of the long rod) can be either 50 mm or 80 mm. For the BiPOWR study, we performed bilateral OWSER implantation, using implants with 50 mm reserve length. We did not perform manual axial trunk traction to achieve lengthening in the outpatient clinic. We instead relied only on passive lengthening due to spinal growth, in combination with normal traction and bending generated through daily activities.

All patients in the BiPOWR trial were allowed unrestricted physical activities post-operatively.

### Outcomes

Demographic parameters such as age, sex, and etiology were recorded as well as surgical parameters such as surgical time and estimated blood loss. Radiographic measurements included main coronal Cobb angle, pelvic obliquity (measured according to the method described by Maloney et al. [37]), T5-T12 kyphosis and L1-S1 lordosis. In addition, we measured T1-T12 and T1-S1 height and -length. Height was calculated as the shortest distance between horizontal lines that were drawn through the midpoint of the upper and lower endplate. For the length measurements, a spline curve was drawn through each endplate that followed the curvature of the spine. These freehand length measurements are less influenced by changes in coronal and sagittal curve [29,38]. All height and length measurements were drawn on both the coronal and sagittal radiographs and the values were averaged. Initial implant growth in the first days after surgery was assessed as the achieved lengthening compared to the maximally loaded spring (SDS) or initial notched rod length (OWSER).

We investigated the occurrence of (serious) adverse events ((S)AEs) in both groups. (S)AEs were classified as disease-related (i.e. related to EOS such as respiratory insufficiency), surgery-related (e.g. surgical site infection) or implant-related (e.g. mechanical failure). For disease- and surgery-related complications we only registered SAEs, i.e. those events which (could) result in permanent disability/damage, or required hospitalization (or lengthening thereof), an unplanned return to the operating room (UPROR), or outpatient medical managing (e.g. antibiotic treatment for SSI). For implant-related complications, all AEs were registered. This includes all complications that were visible on patient radiographs, also if they did not have any apparent clinical consequences (e.g. failure to lengthen), a comprehensive list with criteria for these criteria in “growth-friendly” systems was published previously [30]. To classify severity of (S)AEs, we used the method of Smith et al. [39] In case of (S)AEs, the time of occurrence after initial surgery was also recorded.

All radiographic measurements were performed independently by two researchers (JVCL and CST), using the Surgimap v2.3.2.1 software (Nemaris, New York, USA). Both researchers were blinded to the other's measurements. For all continuous measurements the arithmetic mean of both assessors' measurement was taken. Interrater reliability between both authors, calculated using the intraclass correlation coefficient, was 0.92, indicating excellent reliability. In regard to (S)AEs, the Data Safety and Monitoring Board arbitrated the final decision on presence or absence of (S)AEs in case no consensus was reached.

### Statistics

Baseline characteristics are shown in both groups. Changes in outcome parameters over time are shown as mean (SD) or median (IQR). We employed a mixed repeated-measures ANOVA to determine differences between pre- and post-operative values. Age at surgery and sex were added as covariates. The interaction between follow-up and treatment group was calculated to determine whether the post-operative change differed significantly between groups. The changes from post-operatively until 1-year follow-up were investigated with linear mixed models, to account for missing data, changes in treatment group during follow-up, different follow-up times and to identify the independent effects of several variables. A restricted maximum likelihood estimator model was created for each radiographic variable with sex, age at surgery, pre-operative value, follow-up time and treatment group as fixed effects. In addition, the interaction between follow-up time and treatment group was added as a fixed effect, to identify whether changes over time differed between groups. Patient ID was added as a random effect.

(S)AE rates in both groups were shown as (S)AEs/patient/year. The time to complications between groups were plotted in Kaplan-Meier curves, and proportional hazards were compared with the Mantel-Cox method.

The statistical procedures were performed in IBM SPSS Statistics 28.0 (IBM Corp., Armonk, NY, USA) with the exception of the linear mixed models, which were performed in R Statistical software version 4.0.2 (R Foundation for Statistical Computing, Vienna, Austria) and the survival analysis, which was performed in GraphPad Prism v 10.2.3 (Graphpad Software, San Diego, CA, USA). A *P* < .05 was chosen as statistical significance.

## Results

### Patient inclusions and protocol deviations

**Figure 3:**
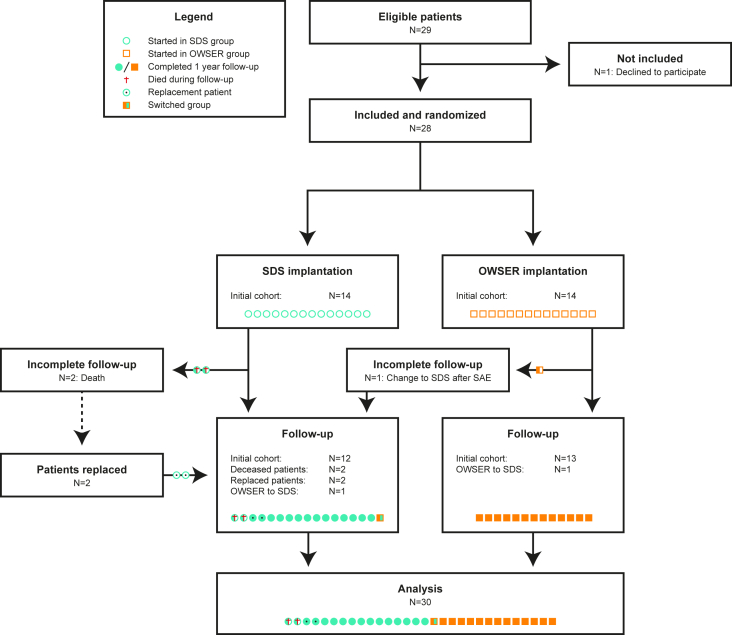
CONSORT patient flow diagram.

During the study period (2019–2023), 30 patients were included, 16 in the SDS group and 14 in the OWSER group. This discrepancy occurred because 2 patients in the SDS group passed away before the end of the 12 month follow-up, due to causes not related to the intervention. These patients were replaced, but the data of all patients was ultimately included in the analysis, up until the point where the patients were lost to follow-up. One of the OWSER patients suffered an SAE that necessitated a re-operation and implant change. The caregivers of this patient had the strong desire that their child receive the SDS implant for the remainder of the study period. Given that a linear mixed model was used for the follow-up analysis (which accurately takes into account the outcome differences if a participant changes from their allocated group), we agreed with this request. This patient was left in the analysis and was followed until the end of the study period. A CONSORT flow diagram of study participants can be seen in Fig. 3.

### Baseline characteristics

Baseline characteristics are reported in Table 2. Mean age of patients at surgery was 9.0 years, 20/30 patients were male. Many different neuromuscular EOS etiologies were included, spinal muscular atrophy was most prevalent. Median surgical time was 180 min, median estimated blood loss was 300 mL and median time to discharge was 6 days.

**Table 2.** Baseline characteristics.

Variable			All patients (N = 30)	SDS patients (N = 16)	OWSER patients (N = 14)
Demographics	Sex (Female)		10/30 (33%)	6/16 (38%)	4/14 (29%)
	Age at surgery (years)		9.0 (SD 1.6)	8.6 (SD 1.5)	9.3 (SD 1.7)
	EOS etiology	SMA type I	3 (10%)	2 (12.5%)	1 (7.1%)
		SMA type II	8 (26.7%)	6 (37.5%)	2 (14.3%)
		Cerebral palsy	2 (6.7%)	1 (6.3%)	1 (7.1%)
		Spina bifida	2 (6.7%)	1 (6.3%)	1 (7.1%)
		Spinal cord injury	2 (6.7%)	1 (6.3%)	1 (7.1%)
		Congenital myopathy	2 (6.7%)	1 (6.3%)	1 (7.1%)
		4q22 syndrome	1 (3.3%)		1 (7.1%)
		KCNQ2 epileptic encephalopathy	1 (3.3%)		1 (7.1%)
		Lennox-Gestaut epileptic encephalopathy	1 (3.3%)	1 (6.3%)	
		Merosin deficient congenital dystrophy 1a	1 (3.3%)	1 (6.3%)	
		Angelman syndrome	1 (3.3%)		1 (7.1%)
		Myelitis transversa	1 (3.3%)		1 (7.1%)
		Noonan syndrome	1 (3.3%)	1 (6.3%)	
		Spastic tetraplegia	1 (3.3%)		1 (7.1%)
		Aicardi-Gourtiers syndrome	1 (3.3%)		1 (7.1%)
		Nemalin myopathy	1 (3.3%)	1 (6.3%)	
		Hydrocephalus	1 (3.3%)		1 (7.1%)
Peri-operative characteristics	Surgical time (minutes)		180 (IQR 64)	170 (IQR 72)	193 (IQR 41)
	Estimated blood loss (mL)		300 (IQR 158)	300 (IQR 150)	320 (IQR 185)
	Time until discharge (days)		6.0 (IQR 2.0)	5.0 (IQR 2.0)	6.5 (IQR 2.0)
Pre-operative measurements	Main coronal Cobb (°)		74.9 (SD 14.7)	77.0 (SD 15.0)	72.4 (SD 14.4)
	Pelvic obliquity (°)		35.5 (SD 14.9)	37.4 (SD 17.4)	33.3 (SD 11.8)
	T5-T12 kyphosis (°)		27.0 (SD 20.9)	21.8 (SD 19.1)	33.5 (SD 22.0)
	L1-S1 lordosis (°)		−37.0 (IQR 29.2)	−28.6 (IQR 26.4)	−46.8 (IQR 28.6)
	T1-T12 height (mm)		179 (SD 25)	172 (SD 22)	188 (SD 26)
	T1-S1 height (mm)		292 (SD 37)	282 (SD 39)	303 (SD 32)
	T1-T12 freehand length (mm)		201 (SD 22)	193 (SD 15)	211 (SD 24)
	T1-S1 freehand length (mm)		331 (SD 36)	319 (SD 30)	344 (SD 38)

SDS, spring distraction system; OWSER, one way self-expanding rod; SMA, spinal muscular atrophy; SD, standard deviation; IQR, interquartile range.

### Curve characteristics

**Figure 4:**
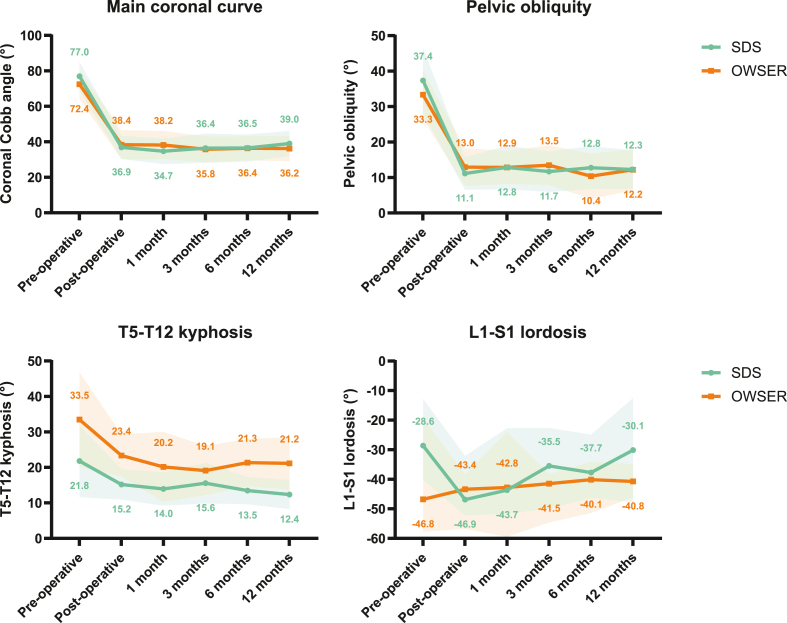
**Coronal- and sagittal curve changes over time**. Mean or median and 95% confidence interval of each timepoint are plotted for each group.

Curve correction results are shown in Table 3, Fig. 4 and Supplement 2. The results of the linear mixed models are shown in Supplement 3. Mean coronal curve decreased from 74.9° pre-operatively, to 37.6° post-operatively and remained stable at 37.7° at 1 year follow-up. In SDS patients, the main coronal curve decreased 52% post-operatively, while at 1 year follow-up, coronal curve correction was 48%. For OWSER, the main coronal curve correction was 47% post-operatively, and 50% at 1 year follow-up. The changes over time were not statistically significant between groups, both for the immediate follow-up (*P* = .128) and at 1 year follow-up (*P* = .180). With respect to pelvic obliquity, both groups showed 60–70% correction post-operatively, which was maintained at 1 year follow-up.

**Table 3.** Changes over time.

		Pre-operative	Post-operative	Post-operative change		*P* value between groups∗	12 months	Follow-up change†		*P* value between groups‡
				Absolute	%			Absolute	%	
Main coronal Cobb (°)	SDS	77.0	36.9	−40.1	−52%	.128	39.0	+2.1	+6%	.180
	OWSER	72.4	38.4	−34.0	−47%		36.2	−2.2	−6%	
Pelvic obliquity (°)	SDS	37.4	11.1	−26.3	−70%	.253	12.3	+1.2	+11%	.162
	OWSER	33.3	13.0	−20.3	−61%		12.2	−0.8	−6%	
T5-T12 kyphosis (°)	SDS	21.8	15.2	−6.6	−30%	.806	12.4	−2.8	−18%	.417
	OWSER	33.5	23.4	−10.1	−30%		21.2	−2.2	−9%	
L1-S1 lordosis (°)	SDS	−28.6	−46.9	−18.3	+64%	.844	−30.1	+16.8	−36%	.776
	OWSER	−46.8	−43.4	+3.4	−7%		−40.8	+2.6	−6%	
T1-T12 freehand length (mm)	SDS	193	207	+14	+7%	.150	225	+18.0	+9%	.295
	OWSER	211	222	+11	+5%		231	+9.0	+4%	
T1-S1 freehand length (mm)	SDS	319	345	+26	+8%	.929	371	+26.0	+8%	.928
	OWSER	344	365	+21	+6%		383	+18.0	+5%	
Cumulative concave implant growth (mm)	SDS	NA	7.9	NA	NA	NA	30.9	+23.0	+291%	.808
	OWSER	NA	4.0	NA	NA		23.2	+19.2	+480%	
Cumulative convex implant growth (mm)	SDS	NA	9.3	NA	NA	NA	29.7	+20.4	+219%	.389
	OWSER	NA	5.8	NA	NA		24.3	+18.5	+319%	

SDS, spring distraction system; OWSER, one way self-expanding rod; NA, not applicable.

∗ *Interaction between time and group in the mixed ANOVA comparing SDS and OWSER groups. Gender and age at surgery are covariates.*

† *Change between 1 year follow-up and post-operatively.*

‡ *Interaction between time and group in the linear mixed model comparing SDS and OWSER groups. Gender, age at surgery and pre-operative values are covariates.*

When comparing T5-T12 kyphosis and L1-S1 lordosis between groups, we observed that SDS patients had a somewhat lower T5-T12 kyphosis and L1-S1 lordosis pre-operatively. L1-S1 lordosis increased substantially (from −28.6° to −46.9°) in the SDS group, although most post-operative radiographs were made in a non-weightbearing position in both groups (in contrast to most other radiographs which were performed in a sitting position). When comparing post-operative to 12-month follow-up in the linear mixed model, changes over time in T5-T12 kyphosis (*P* = .417) and L1-S1 lordosis (*P* = .776) were not different between groups.

### Spinal- and implant growth

**Figure 5:**
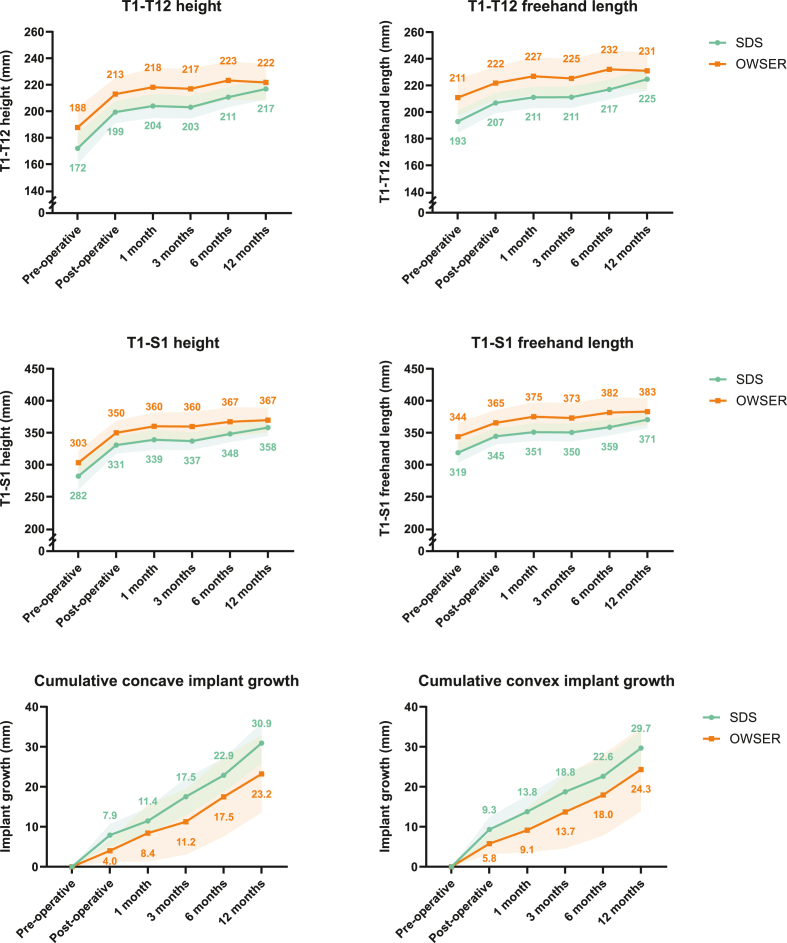
**Spinal height/length changes over time.** Mean and 95% confidence interval of each timepoint are plotted for each group.

Spinal height- and length results can be seen in Table 3, Fig. 5 and Supplement 2. Since OWSER patients were slightly older (and thus taller) at baseline, we looked mainly at the changes between post-operative and 1 year follow-up. The T1-T12 height during 1 year follow-up increased 18 mm in the SDS group, and 9 mm in the OWSER group. For T1-S1 height, growth was 27 mm/year for SDS and 17 mm/year for OWSER.

The freehand length values, which are less influenced by simultaneous changes in coronal or sagittal deformity, similarly showed substantial growth in both groups. The T1-T12 segment increased 18 mm/year for SDS and 9 mm/year for OWSER. For the T1-S1 segment, this was 26 mm/year (SDS) and 18 mm/year (OWSER). The substantial differences between groups disappeared when comparing both groups in the linear mixed model (Supplement 3) showing that both systems support growth of the spine very similarly when correcting for age, sex and pre-operative length. The one-year growth rate following the post-operative phase (1 year follow-up radiograph compared to first post-operative radiograph) was similar for both the concave side of the implant (SDS: 23 mm/year; OWSER: 19 mm/year; *P* = .808) as well as the convex side (SDS: 20 mm/year; OWSER: 19 mm/year; *P* = .389).

Initial implant growth is the rod expansion due to tissue creep that can be seen at the first post-operative radiograph. The SDS spring had already distracted 7.9 mm on the concave side and 9.3 mm on the convex side, compared to 4.0 mm (*P* = .022) and 5.8 mm (*P* = .019) in the OWSER group. This length gain was not included in the growth rate over time.

### (Serious) adverse events

During the study period, 16 (S)AEs were recorded, 5 in the SDS group and 11 in the OWSER group, which corresponded to 0.30 (S)AEs/patient/year in the SDS group, and 0.78 (S)AEs/patient/year in the OWSER group. There was 1 UPROR in the SDS group (0.06/patient/year) and 5 UPRORs in the OWSER group (0.35/patient/year). Especially a higher number of mechanical failures explained this difference (Table 4).

**Table 4.** (S)AE's and UPRORs.

Patient	(S)AE number	(S)AE	(S)AE type	(S)AE severity∗	UPROR	Treatment
SDS 1	1	Superficial SSI	Surgery	I	No	Oral antibiotics
	2	Complete implant extension	Implant	IIa	Yes	Retensioning of spring and lengthening of the growing rod
SDS 2	3	Respiratory insufficiency due to aspiration	Disease	IV	No	None
SDS 3	4	Respiratory insufficiency	Disease	II	No	Prolonged admission
SDS 4	5	Respiratory insufficiency due to accidental uncoupling from breathing equipment	Disease	IV	No	None
OWSER 1	6	Respiratory insufficiency	Disease	II	No	IC admission
OWSER 2	7	OWSER ratchet mechanism failure with implant shortening	Implant	I	No	Expectative
OWSER 3	8	OWSER set screw failure with loss of correction	Implant	IIa	Yes	New OWSER implantation
	9	Complete implant extension	Implant	IIa	Yes	New OWSER implantation
OWSER 4	10	Failure to lengthen	Implant	I	No	Expectative
OWSER 5	11	OWSER ratchet mechanism failure with implant shortening	Implant	IIa	Yes	Revision to SDS treatment
	12	Pneumonia	Disease	II	No	Prolonged admission + antibiotics
OWSER 6	13	Pneumonia	Disease	II	No	Prolonged admission + antibiotics
OWSER 7	14	Deep SSI	Surgery	IIb	Yes	I&D (2x) + vacuum assisted closure + IV and oral antibiotics
OWSER 8	15	Pneumonia	Disease	II	No	Prolonged admission + antibiotics
	16	OWSER endcap loosening	Implant	I	No	Expectative

UPROR, unplanned return to the operating room; SDS, spring distraction system; OWSER, one way self-expanding rod; SSI, surgical site infection; I&D, Irrigation and debridement.

∗ *Disease-related complications: Grade I: Outpatient medical management; Grade II: Inpatient medical management; Grade III: Requires abandoning growth-friendly strategy; Grade IV: Death. Surgery- or implant-related complications: Grade I: Does not require unplanned surgery; Grade IIa: Requires 1 unplanned surgery; Grade IIb: Requires multiple unplanned surgeries; Grade III: Requires abandoning growth-friendly strategy; Grade IV: Death.*

**Figure 6:**
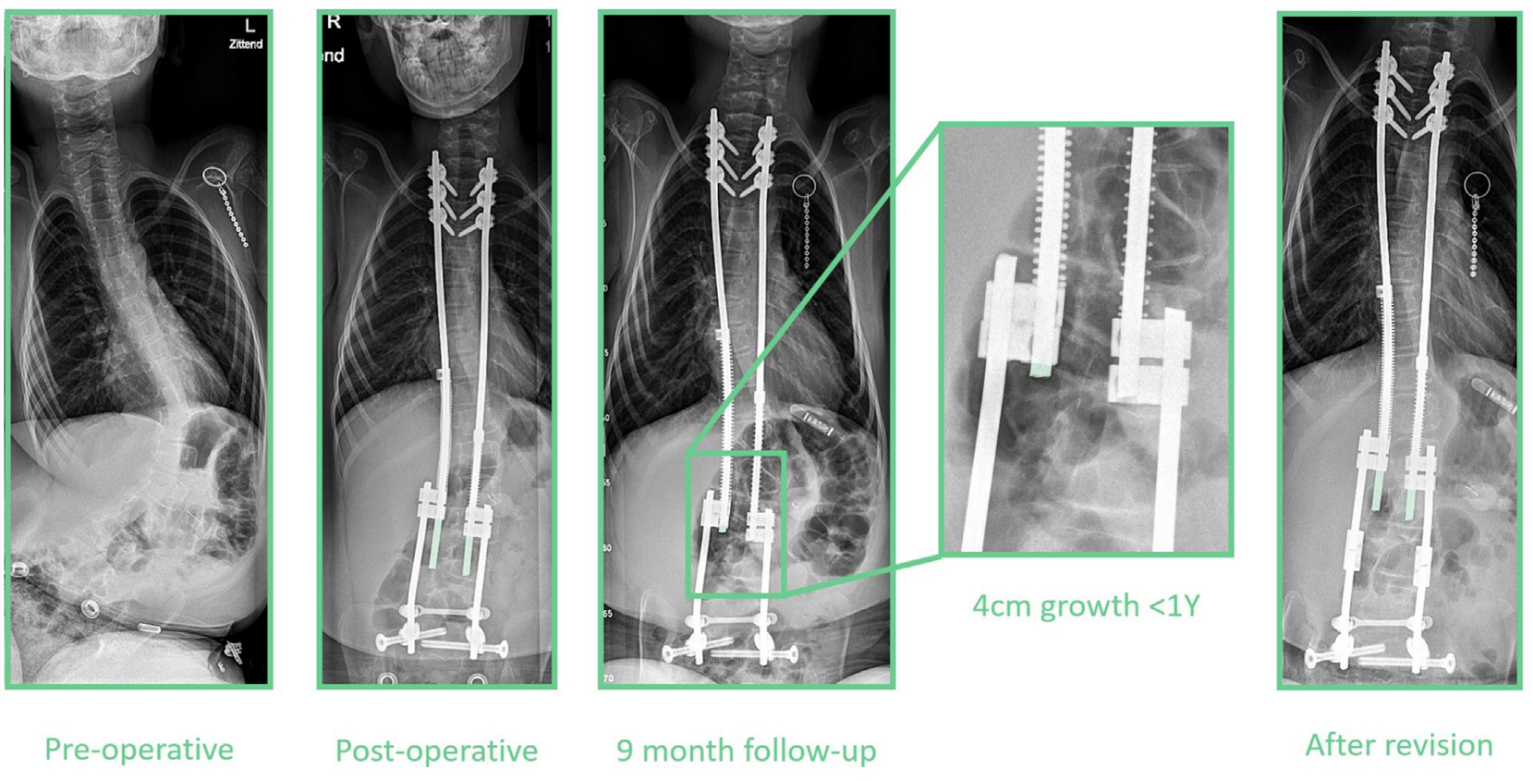
**SDS complication.** Example of an SDS patient that showed almost complete implant expansion within 1 year. Note the initial lengthening between insertion and the first radiograph of 20 mm. As there was still at least 3 years of expected spinal growth, the choice was made to perform a re-operation in which the springs were re-tensioned and the distal anchor rod construct was lengthened. The colored segment of the sliding rod denotes how much implant growth is left.

**Figure 7:**
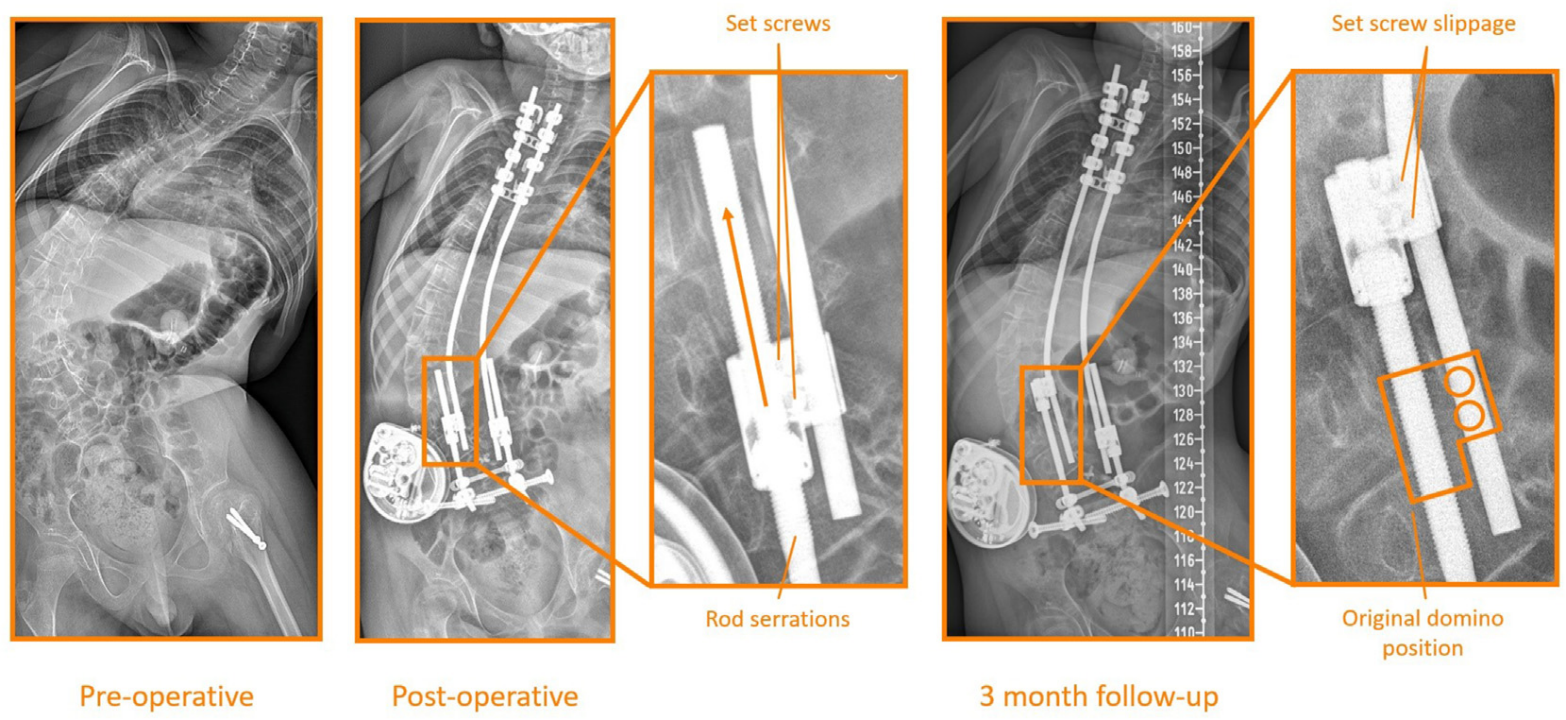
**OWSER complication**. In this patient, the set screws that connect the OWSER domino to the anchor rod failed, causing the domino to erroneously move up on the serrated rod, without achieving any spinal lengthening or additional correction. In this patient, the OWSER rod was replaced during a re-operation.

**Figure 8:**
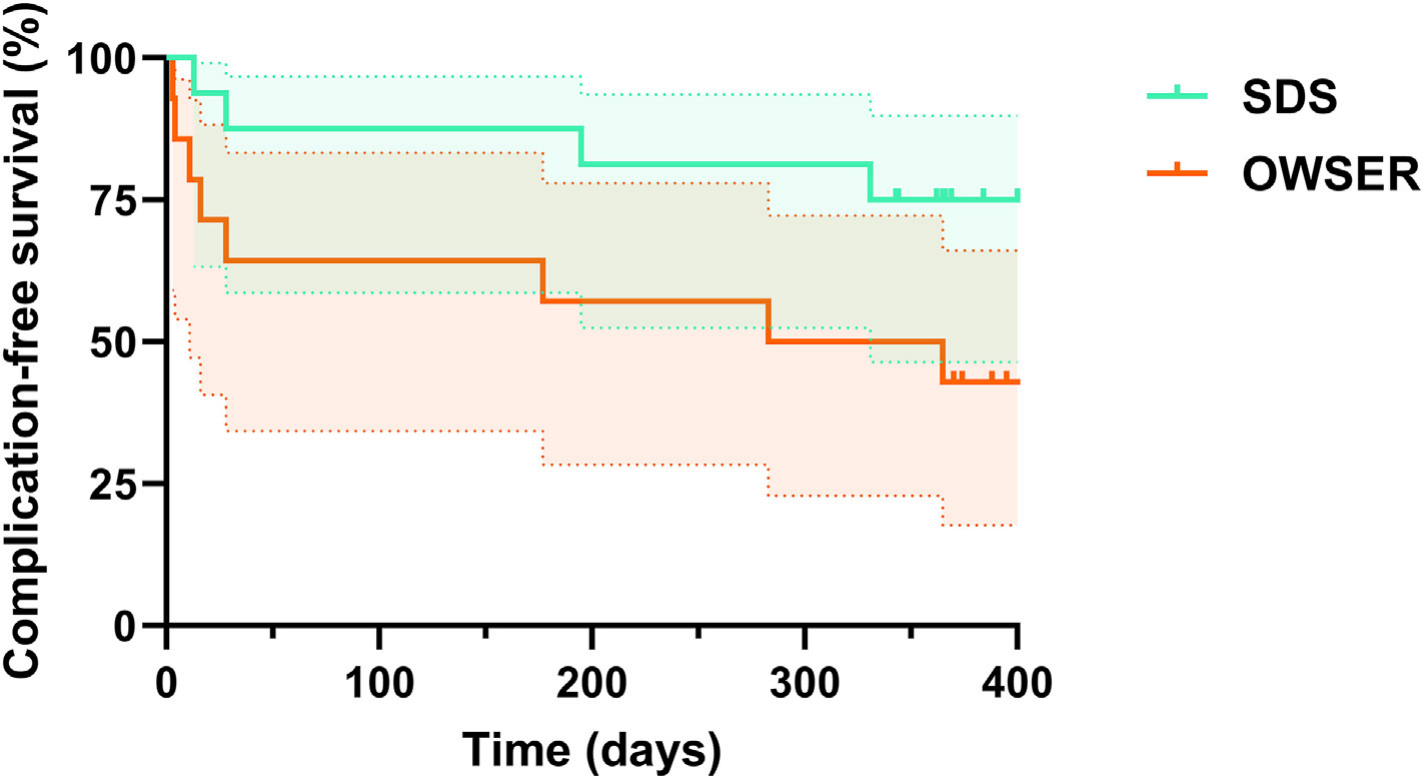
**Kaplan-Meier curves for (S)AE-free survival.** Survival curves for both groups with 95% confidence intervals. Ticks denote censored patients.

In the SDS group, two deaths occurred in the follow-up period between 6 and 12 months post-operatively, when the patient was at home. In both patients, no apparent relation was found between the treatment and the event. One patient aspirated and went into cardiac arrest. The other patient was inadvertently uncoupled from his respiratory equipment, resulting in respiratory insufficiency and cardiac arrest. There were only 2 cases of SSI. One SDS patient suffered a superficial SSI which was treated only with oral antibiotics. One patient in the OWSER group suffered a deep SSI. This patient underwent 2 UPRORs in which irrigation and debridement was performed, followed by vacuum assisted closure of the wound and 12 weeks of antibiotics. Both groups had a patient who showed complete implant expansion within 1 year (Fig. 6). In both patients, expected residual growth was high, and both patients were re-operated; the SDS patient underwent retensioning of the springs, while the OWSER patient received new OWSER rods. In one OWSER patient, the set screws fixating the OWSER domino to the anchor rod failed, which caused complete implant lengthening and loss of correction within 3 month follow-up (Fig. 7). This patient was re-operated and received a new OWSER device. In two other OWSER patients, a failure in the ratchet mechanism occurred, causing loss of correction in one for which the OWSER was replaced. During UPRORs in both groups, some metallosis was observed at the sliding connections.

(S)AE-free survival is graphed in a Kaplan-Meier curve in Fig. 8. The Mantel-Cox test showed a hazard ratio (SDS hazard/OWSER hazard) of 0.34 (95% CI 0.11 to 1.09), however, this was not statistically significant (*P* = .07).

## Discussion

The current study aimed to describe the one-year efficacy and safety of two novel “growth-friendly” implants to surgically treat neuromuscular EOS. Both implants provided 50% curve correction, which could be maintained at 1-year follow-up. Height- and length gain was favorable for both techniques and often exceeded physiological T1-S1 growth (1.5–2.0 cm/year) as described by Dimeglio [40,41]. These values are higher than those seen in MCGR cohorts [[42], [43], [44]]. This may be explained by the fact that SDS and OWSER exhibit continuous, gradual growth. This contrasts the intermittent, forceful distractions often seen in TGR or MCGR, which may cause stiffening of the spine, requiring higher distraction forces and which result in reduced growth over time, known as the “law of diminishing returns” [19,20]. Whether this phenomenon is absent in SDS and OWSER patients is not yet known, and will require longer follow-up.

To allow for comparison among “growth-friendly” techniques in terms of growth we calculated length gain during the “true” growth period (i.e. the period between the first post-operative radiograph and the radiograph at one year [45]. Although the most accurate way to measure spinal length is 3D assessment, this is time consuming and likely not much better than the biplanar method that we used. All measurements indicated that the spinal length gain in the first year was often more than physiological growth, especially in the SDS group. This is likely not accelerated bone growth, but the result of tissue creep and remodeling due to continuous distraction. This opportunity to take advantage of tissue visco-elasticity is a feature of both systems and was clearly demonstrated by the mean initial length gain of 4.9 mm (OWSER) and 8.6 mm (SDS) between surgery and the first post-operative radiograph, due to stretching and spring distraction respectively. The fact that OWSER patients often had a proximal anchor block comprising more levels than SDS patients could have attributed to a relatively small difference in growth over time.

Unfortunately, two patients died during the study. While not ideal in a strict RCT environment, we prospectively decided that in such a situation, the patients could be replaced, as not replacing the patients would have resulted in an underpowered study, which invalidates the results and which would waste the useful study data that was already collected. By replacing these two patients, while also including their results (and the associated SAEs), a fair and balanced analysis between systems could still be performed. These two patient deaths emphasizes the fragility of the neuromuscular EOS population. It confirms that interventions, that can destabilize the fragile balance of these children, especially hospital admission and surgery, should be avoided wherever possible. In that regard, it was encouraging to see that with our bipolar single-surgery approach, only one deep SSI occurred during the study period (3.3%).

A previous meta-analysis in studies including neuromuscular patients showed SSI rates of around 10% [27]. Other studies have shown that the deep infection risk increases with more extensive surgeries and with each subsequent surgery following the initial surgery [13,46]. Depending on the maintenance of correction, it may also be possible to forego definitive spinal fusion, as this “final” surgery carries an additional 40% UPROR risk in neuromuscular patients [47]. Obviously, follow-up until after skeletal maturity is necessary before any conclusions can be drawn on this treatment option.

As expected for this population and procedure, (S)AEs and UPRORs could not be prevented. Especially in the OWSER group, there was a tendency toward implant-related (S)AEs that required UPRORs. Several of these implant failures were related to the ratchet mechanism which apparently is vulnerable and may be improved. Another reason for UPROR was excessive length gain that exceeded the implant capacity within one year. This happened for one patient in both groups and was not related to an anchor- or implant failure, but largely a result of further correction and stretching. As both patients had substantial growth left, we decided to retension/replace the implants. This finding indicates that longer reserve implant lengths are desirable. The OWSER implant already has a version with a lengthening capacity of 80 mm instead of 50 mm. For SDS, the residual rod length can simply be left longer and springs can be stacked [48]. However, there are technical challenges as the extra rod length can protrude, especially in small children.

The BiPOWR study is the first comparative trial that utilizes a randomized design to compare different “growth-friendly” implants for EOS. So far, most systems were investigated retrospectively or in single arm prospective cohort studies, which are prone to confounding (by indication) and (selection) bias, although these issues can be mitigated with sound methodological practices [[49], [50], [51]]. Another way to compensate for such biases is the use of real world data from (large) registries that are published more and more and probably tell us most accurately what is the value of these implants.

A strength of the current study was the strict randomization and blinding design which enabled unbiased comparison between systems in terms of efficacy and safety. While parents often showed initial apprehension when hearing that their child would be randomized to a surgical intervention, we were able to explain the importance of this practice for the validity of the results. Ultimately, all eligible patients, except for one, chose to be included into the trial. Another strength is that all radiographs were systematically measured by the same two authors, reducing measurement variability. (S)AE's were scored using previously reported grading- and classification criteria, which allows for less ambiguity when deciding whether an (S)AE needed to be included.

Limitations of the study include its relatively small cohort with many different neuromuscular diseases. In addition, the follow-up of only 1 year is short and only allows to focus on initial efficacy and safety. Whether the “law of diminishing returns” remains absent for the SDS and OWSER implants is not yet known. This will require much longer follow-up and will be the subject of future research. Only then can we draw conclusions on long-term efficacy and -safety.

## Conclusion

Two self-distracting “growth-friendly” implants were investigated and compared for the treatment of neuromuscular EOS. Both the Spring Distraction System (SDS) and the One Way Self-Expanding Rod (OWSER) achieved coronal curve correction of around 50%, which was maintained at one year follow-up. Spinal length increase was excellent for both systems and partially the result of creep. (Serious) Adverse Events occurred at a relatively low rate in both cohorts, with an unplanned return to the OR rate of 6% for SDS and 35% for OWSER per patient per year.

## Consent for publication

The author(s) declare that no patient consent was necessary as no images or identifying information are included in the article.

## Ethical approval and consent

The current study conforms to the Declaration of Helsinki and was prospectively approved by the Institutional Review Board of UMC Utrecht. All study participants and their parents or legal guardians provided written informed consent before being enrolled into the current study.

## Data availability statement

Research data can be obtained upon reasonable request.

## Author contributions

**Justin V.C. Lemans:** Writing – review & editing, Writing – original draft, Visualization, Project administration, Methodology, Investigation, Formal analysis, Data curation, Conceptualization. **Casper S. Tabeling:** Writing – review & editing, Writing – original draft, Project administration, Methodology, Investigation, Formal analysis, Data curation. **Agnita Stadhouder:** Writing – review & editing, Project administration, Investigation, Formal analysis, Data curation. **Jeroen J.M. Renkens:** Writing – review & editing, Project administration, Investigation, Formal analysis, Data curation. **E. Pauline Scholten:** Writing – review & editing, Project administration, Investigation, Data curation, Conceptualization. **Hilde W. Stempels:** Writing – review & editing, Project administration, Methodology, Investigation, Data curation, Conceptualization. **Lotfi Miladi:** Writing – review & editing, Project administration, Formal analysis. **René M. Castelein:** Writing – review & editing, Supervision, Investigation, Formal analysis, Data curation, Conceptualization. **Moyo C. Kruyt:** Writing – review & editing, Supervision, Methodology, Investigation, Funding acquisition, Formal analysis, Data curation, Conceptualization.

## Funding

This is an investigator initiated study. The UMC Utrecht is responsible for the initiation and management of the clinical trial and is owner of the research data. This study received research funding from Euros (La Ciotat, France). In addition, Euros provided the OWSER devices for use in the current study. The funder had no influence on the design of the study, collection-, analysis- and interpretation of the data, writing of the manuscript, nor on the decision to publish.

## Declaration of competing interests

RMC and MCK are the co-inventors of the SDS, the patent of which is currently held by Cresco Spine. B.V., which aims to valorize the SDS. RMC and MCK are the co-founders of Cresco Spine. LTM is the inventor of the OWSER (which was CE-marked in 2013). The remaining authors declare that they have no competing interests.

## Figures and Tables

**figs1:**