# Supplementary material for: One Year Results of the Randomized BiPOWR Trial Comparing the Spring Distraction System (SDS) and the One Way Self-Expanding Rod (OWSER) for the Correction of Neuromuscular and Syndromic Early Onset Scoliosis
Source: J Pediatr Soc North Am. 2025 Mar 27;11:100180. doi: 10.1016/j.jposna.2025.100180 (PMC12088330; doi:10.1016/j.jposna.2025.100180)
Supplement: Multimedia component 1 [file mmc1.docx]

**Supplement 1: CONSORT Checklist**

| **Section/Topic** | **Item No** | **Checklist item** | **Reported on page No** |
| --- | --- | --- | --- |
| **Title and abstract** |  |  |  |
|  | 1a | Identification as a randomized trial in the title | Title |
|  | 1b | Structured summary of trial design, methods, results, and conclusions (for specific guidance see CONSORT for abstracts) | Abstract |
|  | | | |
| **Introduction** |  |  |  |
| Background and objectives | 2a | Scientific background and explanation of rationale | 3 |
|  | 2b | Specific objectives or hypotheses | 4 |
|  | | | |
| **Methods** |  |  |  |
| Trial design | 3a | Description of trial design (such as parallel, factorial) including allocation ratio | 4 |
|  | 3b | Important changes to methods after trial commencement (such as eligibility criteria), with reasons | NA |
| Participants | 4a | Eligibility criteria for participants | 18 |
|  | 4b | Settings and locations where the data were collected | 4 |
| Interventions | 5 | The interventions for each group with sufficient details to allow replication, including how and when they were actually administered | 4, 5 |
| Outcomes | 6a | Completely defined pre-specified primary and secondary outcome measures, including how and when they were assessed | 4, 5 |
|  | 6b | Any changes to trial outcomes after the trial commenced, with reasons | NA |
| Sample size | 7a | How sample size was determined | 3 |
|  | 7b | When applicable, explanation of any interim analyses and stopping guidelines | NA |
| Randomisation: Sequence generation | 8a | Method used to generate the random allocation sequence | Protocol paper, REF 33 |
|  | 8b | Type of randomisation; details of any restriction (such as blocking and block size) | Protocol paper, REF 33 |
| Randomisation: Allocation concealment mechanism | 9 | Mechanism used to implement the random allocation sequence (such as sequentially numbered containers), describing any steps taken to conceal the sequence until interventions were assigned | 3, Protocol paper, REF 33 |
| Randomisation: Implementation | 10 | Who generated the random allocation sequence, who enrolled participants, and who assigned participants to interventions | Protocol paper, REF 33 |
| Blinding | 11a | If done, who was blinded after assignment to interventions (for example, participants, care providers, those assessing outcomes) and how | 3 |
|  | 11b | If relevant, description of the similarity of interventions | 3, 4 |
| Statistical methods | 12a | Statistical methods used to compare groups for primary and secondary outcomes | 5 |
|  | 12b | Methods for additional analyses, such as subgroup analyses and adjusted analyses | 4, 5 |
|  | | | |
| **Results** |  |  |  |
| Participant flow (a diagram is strongly recommended) | 13a | For each group, the numbers of participants who were randomly assigned, received intended treatment, and were analysed for the primary outcome | Figure 3 |
|  | 13b | For each group, losses and exclusions after randomisation, together with reasons | Figure 3 |
| Recruitment | 14a | Dates defining the periods of recruitment and follow-up | 5 |
|  | 14b | Why the trial ended or was stopped | NA |
| Baseline data | 15 | A table showing baseline demographic and clinical characteristics for each group | Table 2 |
| Numbers analysed | 16 | For each group, number of participants (denominator) included in each analysis and whether the analysis was by original assigned groups | 5, 6, Figure 3 |
| Outcomes and estimation | 17a | For each primary and secondary outcome, results for each group, and the estimated effect size and its precision (such as 95% confidence interval) | Table 3, Table 4, Figure 4, Figure 5, Supplement 2, Supplement 3 |
|  | 17b | For binary outcomes, presentation of both absolute and relative effect sizes is recommended | NA |
| Ancillary analyses | 18 | Results of any other analyses performed, including subgroup analyses and adjusted analyses, distinguishing pre-specified from exploratory | 6, 7, Figure 8, Supplement 3 |
| Harms | 19 | All-important harms or unintended effects in each group (for specific guidance see CONSORT for harms) | 7, Figure 8 |
|  | | | |
| **Discussion** |  |  |  |
| Limitations | 20 | Trial limitations, addressing sources of potential bias, imprecision, and, if relevant, multiplicity of analyses | 10 |
| Generalisability | 21 | Generalisability (external validity, applicability) of the trial findings | 9, 10 |
| Interpretation | 22 | Interpretation consistent with results, balancing benefits and harms, and considering other relevant evidence | 9, 10 |
|  | | | |
| **Other information** |  |  |  |
| Registration | 23 | Registration number and name of trial registry | 4 |
| Protocol | 24 | Where the full trial protocol can be accessed, if available | 4 |
| Funding | 25 | Sources of funding and other support (such as supply of drugs), role of funders | 10, 11 |
