# Supplementary material for: One Year Results of the Randomized BiPOWR Trial Comparing the Spring Distraction System (SDS) and the One Way Self-Expanding Rod (OWSER) for the Correction of Neuromuscular and Syndromic Early Onset Scoliosis
Source: J Pediatr Soc North Am. 2025 Mar 27;11:100180. doi: 10.1016/j.jposna.2025.100180 (PMC12088330; doi:10.1016/j.jposna.2025.100180)
Supplement: Multimedia component 2 [file mmc2.docx]

**Supplement 2: Curve characteristics and spinal height and length over time**

|  | | **Pre-operative** | **Postoperative** | **1 month** | **3 months** | **6 months** | **12 months** |
| --- | --- | --- | --- | --- | --- | --- | --- |
| Main coronal Cobb (°) | All patients | 74.9 (SD 14.7) | 37.6 (SD 13.0) | 36.3 (SD 12.3) | 36.1 (SD 12.7) | 36.4 (SD 12.3) | 37.7 (SD 12.2) |
|  | SDS | 77.0 (SD 15.0) | 36.9 (SD 12.3) | 34.7 (SD 12.5) | 36.4 (SD 14.2) | 36.5 (SD 13.8) | 39.0 (SD 12.8) |
|  | OWSER | 72.4 (SD 14.4) | 38.4 (SD 14.1) | 38.2 (SD 12.4) | 35.8 (SD 11.4) | 36.4 (SD 10.7) | 36.2 (SD 11.8) |
| Pelvic  obliquity (°) | All patients | 35.5 (SD 14.9) | 12.0 (SD 8.8) | 12.9 (SD 9.1) | 12.5 (SD 9.3) | 11.7 (SD 10.4) | 12.2 (SD 9.5) |
|  | SDS | 37.4 (SD 17.4) | 11.1 (SD 8.6) | 12.8 (SD 10.6) | 11.7 (SD 9.9) | 12.8 (SD 10.9) | 12.3 (SD 9.8) |
|  | OWSER | 33.3 (SD 11.8) | 13.0 (SD 9.2) | 12.9 (SD 7.3) | 13.5 (SD 8.8) | 10.4 (SD 10.0) | 12.2 (SD 9.6) |
| T5-T12  kyphosis (°) | All patients | 27.0 (SD 20.9) | 19.0 (SD 9.9) | 16.2 (SD 9.8) | 17.0 (SD 9.8) | 16.8 (SD 9.0) | 16.6 (SD 10.2) |
|  | SDS | 21.8 (SD 19.1) | 15.2 (SD 8.0) | 14.0 (SD 8.2) | 15.6 (SD 9.9) | 13.5 (SD 6.9) | 12.4 (SD 6.7) |
|  | OWSER | 33.5 (SD 22.0) | 23.4 (SD 10.4) | 20.2 (SD 11.8) | 19.1 (SD 9.7) | 21.3 (SD 9.9) | 21.2 (SD 11.6) |
| L1-S1  lordosis (°) | All patients | -37.0 (IQR 29.2) | -44.8 (IQR 14.3) | -43.7 (IQR 21.8) | -38.6 (IQR 19.8) | -39.9 (IQR 16.8) | -37.9 (IQR 20.0) |
|  | SDS | -28.6 (IQR 26.4) | -46.9 (IQR 19.7) | -43.7 (IQR 23.8) | -35.5 (IQR 18.8) | -37.7 (IQR 21.4) | -30.1 (IQR 28.5) |
|  | OWSER | -46.8 (IQR 28.6) | -43.4 (IQR 15.2) | -42.8 (IQR 19.3) | -41.5 (IQR 13.0) | -40.1 (IQR 9.1) | -40.8 (IQR 10.8) |
| T1-T12  height (mm) | All patients | 179 (SD 25) | 206 (SD 19) | 210 (SD 20) | 209 (SD 20) | 216 (SD 19) | 219 (SD 19) |
|  | SDS | 172 (SD 22) | 199 (SD 16) | 204 (SD 16) | 203 (SD 15) | 211 (SD 14) | 217 (SD 14) |
|  | OWSER | 188 (SD 26) | 213 (SD 21) | 218 (SD 23) | 217 (SD 24) | 223 (SD 23) | 222 (SD 23) |
| T1-T12 freehand length (mm) | All patients | 201 (SD 22) | 214 (SD 19) | 218 (SD 20) | 218 (SD 20) | 224 (SD 19) | 228 (SD 19) |
|  | SDS | 193 (SD 15) | 207 (SD 14) | 211 (SD 14) | 211 (SD 14) | 217 (SD 13) | 225 (SD 15) |
|  | OWSER | 211 (SD 24) | 222 (SD 21) | 227 (SD 22) | 225 (SD 23) | 232 (SD 22) | 231 (SD 23) |
| T1-S1  height (mm) | All patients | 292 (SD 37) | 340 (SD 30) | 349 (SD 32) | 347 (SD 31) | 357 (SD 31) | 363 (SD 28) |
|  | SDS | 282 (SD 39) | 331 (SD 25) | 339 (SD 28) | 337 (SD 24) | 348 (SD 23) | 358 (SD 23) |
|  | OWSER | 303 (SD 32) | 350 (SD 33) | 360 (SD 34) | 360 (SD 36) | 367 (SD 36) | 367 (SD 36) |
| T1-S1 freehand length (mm) | All patients | 331 (SD 36) | 354 (SD 31) | 362 (SD 32) | 361 (SD 32) | 369 (SD 32) | 376 (SD 30) |
|  | SDS | 319 (SD 30) | 345 (SD 23) | 351 (SD 24) | 350 (SD 24) | 359 (SD 23) | 371 (SD 24) |
|  | OWSER | 344 (SD 38) | 365 (SD 35) | 375 (SD 35) | 373 (SD 37) | 382 (SD 38) | 383 (SD 35) |
| Cumulative concave implant growth (mm) | All patients | NA | 6.1 (SD 5.3) | 10.0 (SD 8.6) | 14.6 (SD 10.9) | 20.5 (SD 11.9) | 27.3 (SD 13.2) |
|  | SDS |  | 7.9 (SD 5.2) | 11.4 (SD 5.8) | 17.5 (SD 8.1) | 22.9 (SD 7.6) | 30.9 (SD 9.2) |
|  | OWSER |  | 4.0 (SD 4.7) | 8.4 (SD 11.1) | 11.2 (SD 12.9) | 17.5 (SD 15.6) | 23.2 (SD 16.0) |
| Cumulative convex implant growth (mm) | All patients | NA | 7.7 (SD 5.5) | 11.6 (SD 8.1) | 16.4 (SD 11.5) | 20.5 (SD 12.0) | 27.2 (SD 13.1) |
|  | SDS |  | 9.3 (SD 5.8) | 13.8 (SD 7.1) | 18.8 (SD 8.1) | 22.6 (SD 7.6) | 29.7 (SD 7.8) |
|  | OWSER |  | 5.8 (SD 4.6) | 9.1 (SD 8.7) | 13.7 (SD 14.4) | 18.0 (SD 16.0) | 24.3 (SD 17.3) |
| SDS: spring distraction system; OWSER: one way self-expanding rod; NA: not applicable; SD: standard deviation; IQR: interquartile range | | | | | | | |
